# Cyclic Deformation and Correspondent Crack Initiation at Low-Stress Amplitudes in Mg–Gd–Y–Zr Alloy

**DOI:** 10.3390/ma11122429

**Published:** 2018-11-30

**Authors:** Chao He, Yujuan Wu, Liming Peng, Ning Su, Xue Li, Kun Yang, Yongjie Liu, Shucheng Yuan, Renhui Tian

**Affiliations:** 1 School of Architecture and Civil Engineering, Chengdu University, Chengdu 610105, China; hechao@cdu.edu.cn; 2 Key Laboratory of Deep Earth Science and Engineering, Ministry of Education, Sichuan University, Chengdu 610065, China; lixuezi89@163.com (X.L.); scu_yangkun@163.com (K.Y.); yuansc2012@163.com (S.Y.); renhui_tian@126.com (R.T.); 3 National Engineering Research Center of Light Alloy Net Forming and State Key Laboratory of Metal Matrix Composite, Shanghai Jiao Tong University, Shanghai 200240, China; plm616@sjtu.edu.cn (L.P.); ningsu@sjtu.edu.cn (N.S.)

**Keywords:** fatigue crack initiation, cyclic deformation, basal slip, magnesium alloy, high cycle fatigue

## Abstract

Cyclic deformation at low-stress amplitudes of a rare earth-containing magnesium alloy (Mg–Gd–Y–Zr) was investigated with emphasis on the responsible microstructural relationship between deformation mechanism and fatigue crack initiation. The results show that the microstructural deformation is extremely inhomogeneous at the low-stress amplitudes. Both deformation twinning and non-basal slip are barely observed, and basal slip is the predominant deformation to accommodate micro-plasticity. Fatigue crack initiation occurred at the basal slip bands, causing the morphology of facet on the fracture surface. Therefore, the basal slip is of prime importance in low-stress cyclic deformation and fatigue failure, and fatigue improvement could potentially be obtained through hindering the motion of basal dislocation by microstructural obstacles.

## 1. Introduction

Magnesium alloy is an attractive structural material that can be potentially applied in automobile and aerospace industries, due to its low density. However, its strength is relatively low as compared with aluminum alloys. The addition of rare-elements (RE) in Mg alloys could improve the strength remarkably and tailor the deformation mechanisms [1]. The structural materials applied in transportation industries would inevitable working in cyclic loading for a long time during its working service, which results in underlying fatigue failure in engineering application. Therefore, it is necessary to fully understand high cycle fatigue behaviors of rare-elements containing magnesium (Mg–RE) alloys to ensure the structural security and reliability.

It was known that a limited number of independent slip system is available in hexagonal close-packed (hcp) structure at room temperature. The deformation of Mg alloy is generally accompanied by dislocation slip and twinning [2,3]. As compared with conventional Mg alloy, it has been observed that Mg–RE alloy exhibited a higher extent of non-basal slip and deformation twinning, due to its random textures during hot extrusion. Besides, it has been reported that dislocation slip was predominant at low strain amplitudes (0.3%–0.6%) in high cycle fatigue, while twinning governed deformation at high strain amplitudes (0.7%–5.0%) in low cycle fatigue [4,5,6]. In practical applications, structural materials generally deform at stresses much lower than their yield strength, indicating that the corresponding maximum strain is relatively lower than those applied in previous studies. However, the cyclic deformation mechanisms at low strain amplitudes, which is corresponding to the loads in high cycle fatigue regime, were barely investigated. Furthermore, a close relationship between the cyclic deformation mechanisms and fatigue crack initiation has been confirmed according to the studies by Yang et al. [7,8]. Both deformation twinning and slip bands can initiate fatigue crack and lead to final fatigue failure. As for the high cycle fatigue, the cyclic deformations at low-stress amplitudes is still unclear, leading to the ambiguity of the interaction between microstructural deformation and small fatigue crack behaviors.

This work aims at investigating the cyclic deformation mechanisms at low-stress amplitudes responsible for Mg–RE alloy and unraveling its relation to the fatigue crack initiation mechanisms. The research results of this study may provide a deep understanding of high cycle fatigue failure in rare earth-containing Mg alloys.

## 2. Material and Experimental Methods

**Figure 1:**
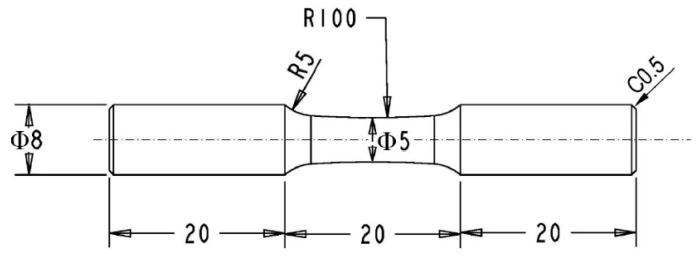
Specimen dimension in rotating bending fatigue testing.

The material used in this study was an extruded magnesium alloy with a diameter of 10 mm. The chemical composition (wt %) is 10Gd, 3Y, 0.5Zr and Mg in balance. The tensile test (Shimazu Inc., Tokyo, Japan) was conducted at room temperature according to the GBT228.1-2010 test standard (Chinese Standard Method). The yield and ultimate strengths of this alloy are 203.8 MPa and 258.1 MPa, respectively. The elongation at the final fracture is 11.5%. In fatigue testing (Shimazu Inc., Tokyo, Japan) dimensions of the specimen are presented in Figure 1, with its axis parallel to the extrusion direction. Rotating fatigue testing system was applied in cyclic deformation, in which the applied stress was defined as the maximum stress across the section [9,10]. The central section was mechanically polished with sandpaper with grids of #2000 and #3000, respectively, then chemically etched in a solution of nitric acid alcohol (volume ratio of 9:1) to reveal the microstructure. To observe the interaction between microstructure and crack behavior, the replication technique was applied to record the fatigue crack initiation process at intervals during fatigue testing. Stress amplitudes at 110 MPa, 120 MPa and 130 MPa, which were equal to the strains of 0.24%, 0.266% and 0.288% respectively, were selected in fatigue testing. The fatigue lives of three specimens are listed in Table 1. After the final failure, fracture surface of the specimen was analyzed with scanning electron microscopy (SEM, ZEISS Inc., Oberkochen, Germany). After fatigue testing, the specimen surface was electrolytically polished to remove the oxide layer formed during testing and slightly etched again to reveal grain boundaries and slip bands. Then, the crystallographic texture of a surface zone was characterized by electron backscatter diffraction (EBSD, EDAX Inc., New Jersey, NJ, USA) technique (TSL OIM analysis 7.2). According to the size of the scanning area, step sizes of 0.2~0.5 μm were used in EBSD orientation image mapping.

## 3. Results and Discussions

### 3.1. Microstructure and Texture

**Figure 2:**
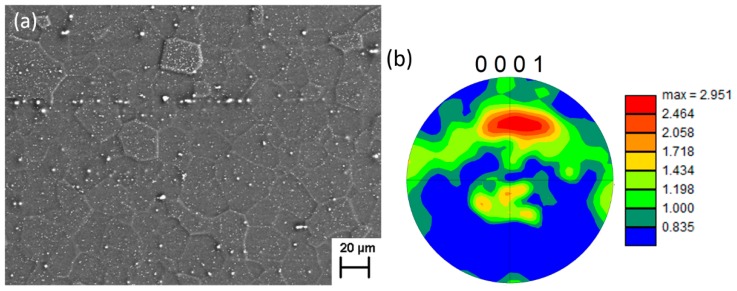
Microstructure at specimen surface along the extrusion direction (**a**) and (0001) Pole figure indicating a relatively randomized texture (**b**).

Figure 2a shows the microstructure at specimen surface along the extrusion direction, in which it consists of equiaxed grains an average size of 32.4 ± 14 μm according to the linear intercept technique. The grains were much finer as compared with conventional Mg alloys, due to the addition of Zr elements [10]. For conventional Mg alloys, it is well known that the texture resulting from the extrusion process has strong influences on their mechanical properties. A significant fraction of grains prefers to orient their c-axis perpendicularly to the extrusion direction, resulting in a strong texture [11]. In Mg–RE alloys, a relatively randomized texture in Figure 2b was obtained with a maximum intensity of 2.951. The addition of solute elements (Gd, Y and Zr) reduced the overall texture sharpness or intensity by hindering deformation twinning, this promoted non-basal slip and refining grain [12]. This randomized texture could effectively remit directional anisotropy and thereby improve the cyclic deformation of extruded Mg alloys.

### 3.2. Cyclic Deformation at Low-Stress Amplitudes

It should be noted that the selected zone of EBSD analysis is about 1.0 mm away from the fracture surface, so the obtained results were caused by cyclic deformation instead of crack propagation-induced deformation near the fracture surface.

**Figure 3:**
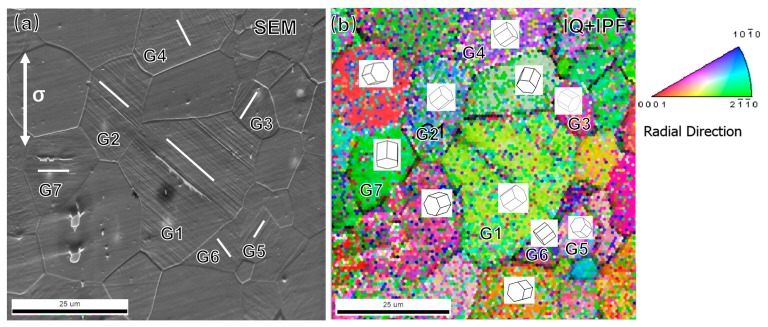
Cyclic deformation mechanisms at a stress amplitude of 130 MPa. (**a**) Slip bands at specimen surface; (**b**) Combined mode of image quality (IQ) and inverse pole figure (IPF) in (**a**).

Figure 3 shows the surface morphology after fatigue testing at 130 MPa and correspondent grain orientation obtained by the EBSD technique. It should be noted that many noises existed in Figure 3b, the lattice orientation of a grain was determined by the highest value of confidence index (CI). For better illustration, typical grains with slip markings were numbered in Figure 3a and the insets show the crystal orientations of these grains in Figure 3b. Surface slip bands can be clearly characterized in the interior of the numbered grains, in which the direction of slip markings was plotted as a short white line in Figure 3a. It is interesting that the direction of slip bands is perfectly parallel to their basal planes of the corresponding lattice. Therefore, the basal slip was activated to accommodate the shear deformation within the numbered grains. In previous studies, cyclic deformation of Mg–Gd–Y alloy has been investigated at the different strain amplitudes. The results showed that a critical strain amplitude (around 0.75%) existed according to the changes of predominant deformation mechanisms from deformation twinning to slipping. However, the activated slip system was still unclear in previous studies. The results in Figure 3 furtherly demonstrated that <a> dislocation has been activated at low-stress amplitudes, but deformation twinning and non-basal slip are barely observed. The limited activated slip system resulted in inhomogeneous microstructural deformations in the microstructure with randomized texture. As a result, the grains with unfavorable orientations for basal dislocation motion would probably be free of slip bands. On the other hand, critical resolved shear stress (CRSS) of <c + a> dislocation in magnesium is generally much higher than that of <a> dislocation by almost two orders of magnitude [13]. The addition of Gd and Y elements would improve the deformation compatibility by decreasing the CRSS of <c + a> dislocation and increasing that of <a> dislocations [14], so the relative difference between CRSS values of the different modes would be reduced to some extent. However, the improvement of deformation compatibility seems to be limited at low-stress amplitudes, because <c + a> dislocations was barely activated according to our observation. The deformation was accommodated mainly by basal slip within isolated grains. Therefore, as the decreasing of applied stress or strain, the deformation heterogeneities become more remarkable, due to the limited slip systems. <a> dislocation prefer to be activated instead of non-basal slip at the stress amplitudes below 130 MPa in polycrystalline Mg alloys.

### 3.3. Fatigue Crack Initiation Mechanism

**Figure 4:**
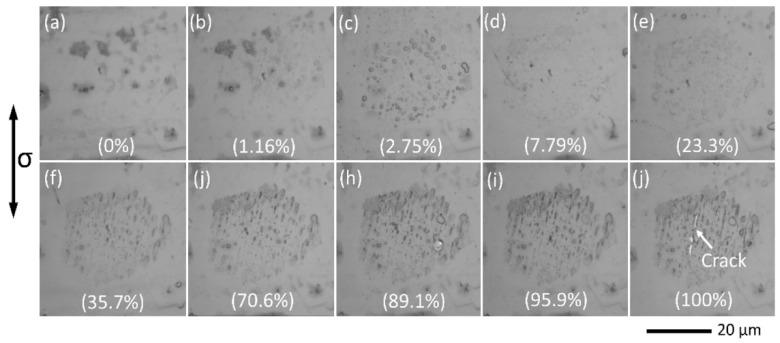
Fatigue crack initiation process at surface slip bands.

Figure 4 shows the evolution process of slip markings within a grain on the specimen surface until the initiation of a secondary crack from the slip bands (120 MPa, 3.51 × 10^5^ cycles). The ratio of the cyclic number (Nc) to total fatigue life (Nf) is inserted within each image. The grain surface became a bit rough almost from the beginning of fatigue test (1.16%), due to the activation of basal slip. Along with the accumulation of micro-plasticity, the grain was filled with slip markings. Finally, a crack initiated from the slip bands as indicated in Figure 4j. The fatigue crack initiation resulted in the cracking of grain along slip bands. Furthermore, it can be observed that the evolution of slip markings is strictly limited within the interior of grain, and the grain boundary could be clearly identified by the saturation of slip bands at the end of fatigue testing. Meanwhile, neighboring grains kept in elastic deformation during the whole period. The plasticity localization at 120 MPa is more remarkable than that at 130 MPa, as shown in Figure 3. Therefore, as the decreasing of applied stress, a limited number of grains could be slip-activated, due to its lower resolved shear stress of basal slip system. As a result, the slip markings were observed at isolated grains with favorable orientation for the basal slip.

**Figure 5:**
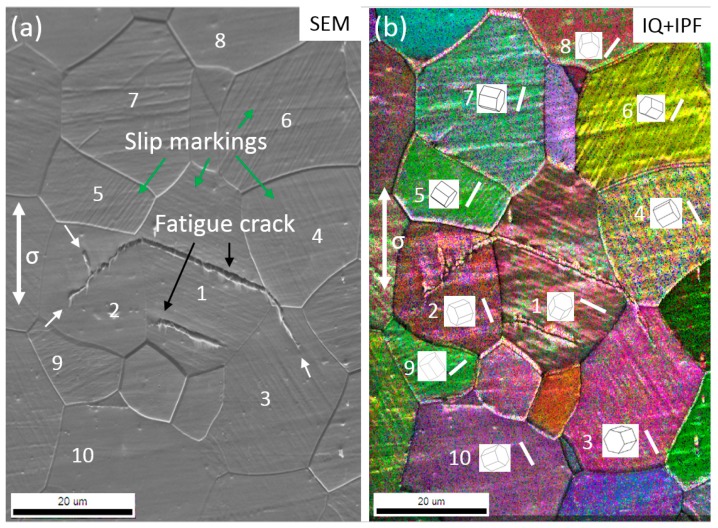
Color-coded orientation map at the fatigued specimen surface (130 MPa): (**a**) SEM image of specimen surface containing a fatigue crack and (**b**) grain orientation of the region in (a).

In order to obtain the crystallographic orientation of the crack initiation site, an area containing secondary cracks on the surface of the specimen at 130 MPa was analyzed by EBSD, as shown in Figure 5. In the SEM image, slip markings are visible at the surface, especially in coarse grains. Two fatigue cracks initiated along the slip markings in the interior of Grain 1. The propagation of the below one was blocked by the grain boundary and the upper one stopped propagating in the interior of its neighboring grains. In the combined modes of IQ and IPF, it was confirmed again that almost all the slip markings agreed with the basal plane as indicated by the orientation of crystal lattice. The fatigue crack paths within the Grains 1 and 3 were parallel to the basal plane. This observation provided direct evidence that basal slip was involved in the formation of the fatigue crack. In Grain 2, the basal plane was approximately perpendicular to the crack path, so the crack tip was branched during the propagation as indicated by white arrows in Figure 5. The bifurcated crack on the upper side was also parallel to the slip bands. Therefore, the fatigue crack in this alloy preferred propagating along the basal slip bands to other directions.

**Figure 6:**
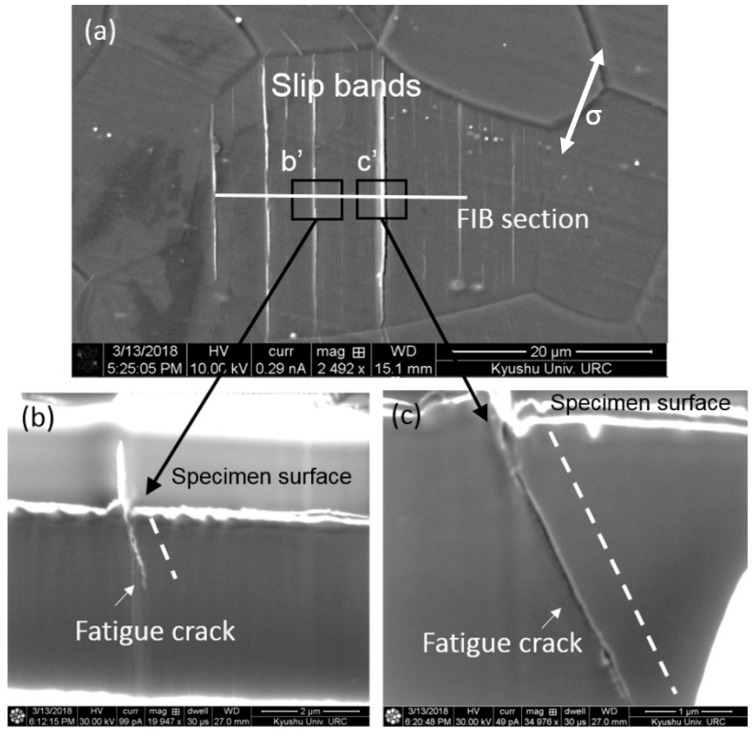
Micrograph of cross-sectioned slip bands by focus ion beam at 130 MPa. (**a**) Slip-induced fatigue cracking at specimen surface; (**b**) observation on the cross section of the slip band as plotted in (**a**); (**c**) straight crack path beneath the specimen surface.

After the crack initiation along slip bands, it would propagate from the surface into the matrix. To gain further understandings of crack behaviors beneath the surface, focus ion beam (FIB) technique was used to section the slip-induced crack. Figure 6 shows the cross-section of the relationship between the slip bands and crack path. Slip-induced cracks could be observed and they were parallelly distributed in the interior of a grain. The white line plotted in Figure 6a indicated the section location by the FIB. The cross-section of location b’ in Figure 6a is shown in Figure 6c, in which a fatigue crack was initiated from the root of intrusion at specimen surface. Generally, the dislocations within slip bands could be released at the surface, forming extrusion and intrusion [15,16]. As a result, the evolution of slip bands resulted in surface roughening during fatigue testing. Over an increasing number of cycles, the stress concentration and extrusion height increase assisting in crack initiation at slip bands [17]. Another example of a sectioned slip band is shown in Figure 6c. surface slip-induced crack initiation was confirmed again, and the crack path beneath the surface is very straight. Besides, the direction of fatigue cracks both in Figure 6b,c was consistently oriented as indicated by white dash lines. Since basal slip was predominant in cyclic deformation as we observed in Figure 3 and Figure 5. It can be deduced that fatigue crack preferred to propagate along the basal plane after the initiation at surface slip markings.

**Figure 7:**
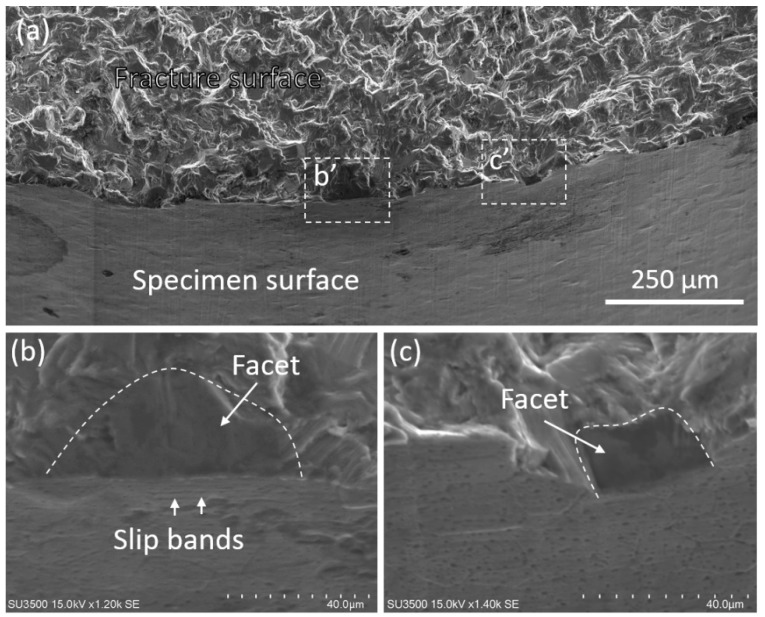
Fracture surface showing the morphology of facet. (**a**) Side view of overall fracture surface; (**b**) and (**c**) facets along fatigue crack path.

The final fracture surface was observed by SEM to investigate fatigue failure mechanisms. Figure 7 shows a typical fracture surface of the specimen tested at 130 MPa. At the edge between fracture surface and specimen surface, numerous facets on the fracture surface were observed, as shown in enlarged view in Figure 7b,c. The existence of facets agreed with the straight crack path after crack initiation along the basal slip bands, as shown in Figure 6. Similar crack nucleation features in high cycle fatigue were also observed in hcp materials, such as magnesium alloys [18,19,20] and titanium alloys [21,22]. The cyclic dislocation motion along the basal plane caused the crack initiation at the surface and the propagation along the slip plane. It can be deduced that the facet preferred to occur at the grains with higher resolved shear stresses along the basal plane. The facets along the slip plane will be initiated during fatigue testing once the shear stress exceeded the CRSS value. Furthermore, the size of facets is generally larger than the average grain size. Therefore, coarse grains can initiate fatigue crack more easily, as a result, a higher probability of localized damage was expected as compared to the fine grains [19].

At low-stress amplitudes, the fatigue limit of Mg–Gd–Y alloy is in the range of 100~120 MPa [4,7,23], indicating that there is no significant improvement as compared with RE-free Mg alloys [8,18,24]. The strengthening mechanisms of static performance seem to be inefficient. The addition of Gd and Y is capable of hindering deformation twinning and promoting non-basal slip, leading to the improvement of mechanical symmetry via texture randomization. However, the applied stress in high cycle fatigue is much lower than the CRSS of non-basal slip in polycrystalline Mg alloy [25], so the non-basal slip is hard to be activated at low-stress amplitudes and is unable to evidently influence the alloy’s high cycle fatigue strength. Based on our study, dislocation slip along basal plane contributes to the deformation of Mg alloys in high cycle fatigue regime, therefore a significant increase of fatigue strength could be expected if the dislocation motion was impeded by some kinds of barriers. For example, introducing precipitates by appropriate heat treatments or interfaces by pre-deformation may prohibit the <a> dislocation motion and bring in an increase of fatigue limit. Our further work will focus on the interfacial design of Mg alloys to improve its high cycle fatigue strength.

## 4. Conclusions

Cyclic deformation mechanism of a rare earth-containing Mg alloy at low-stress amplitudes was investigated for further understanding of high cycle fatigue crack initiation. The following main conclusions can be drawn:

1. At the stress amplitudes below 130 MPa, both twinning and non-basal slip were barely activated. Plastic deformation is mainly accommodated by basal slip. This could be ascribed to increased critical resolved shear stress in polycrystalline Mg alloys;
2. Low-stress deformation in Mg alloy was extremely inhomogeneous, due to its limited slip systems. Slip markings were restricted within isolated grains that have favorable orientation for the basal slip;
3. Fatigue cracks always initiated along surface slip bands, and then propagated into the matrix straightly along basal plane, resulting in facet morphologies at fracture surface;
4. Since basal slip was responsible for predominant deformation and fatigue crack initiation, introducing of specific microstructure or interfaces that are capable of hindering <a> dislocation may significantly improve high cycle fatigue strength.

## Figures and Tables

**Table 1:** Fatigue testing results at three stress amplitudes.

Maximum Stress	Maximum Strain	Fatigue Life
110 MPa	0.244%	No failure
120 MPa	0.266%	3.51 × 10^5^ cycles
130 MPa	0.288%	2.39 × 10^4^ cycles
